# A qualitative analysis of dental challenges for oncology patients receiving bone-modifying agents

**DOI:** 10.1007/s00520-026-10951-0

**Published:** 2026-07-24

**Authors:** Byrne H., Weadick C. S., O.’Reilly S., Ní Ríordáin R.

**Affiliations:** 1 Cork University Dental School and Hospital, Cork City, Cork, T12E8YV Ireland; 2 Cancer Research @UCC, College of Medicine and Health, University College Cork, Cork City, Cork, Ireland; 3 Department of Medical Oncology, Cork University Hospital, Wilton, Cork City, Cork, Ireland

**Keywords:** Supportive care, Onco-dental interface, Medication-related osteonecrosis of the jaw

## Abstract

**Background:**

Bone-modifying agents (BMAs) play an integral role in cancer care treatments. Their interface with dentistry remains challenging for oncology patients receiving BMAs and their general dental practitioners (GDPs) providing care for them. This article uses qualitative research methods to explore the challenges of dental care for patients and GDPs receiving BMAs.

**Methods:**

The study recruited 10 oncology patients receiving BMAs and 20 GDPs. The oncology patients participated in a focus group (*n* = 6) or qualitative interviews (*n* = 4) via telephone interviews. GDPs participated in three separate focus groups (*n* = 20). The interactions were recorded and transcribed, and thematic analysis was completed.

**Results:**

Analyses of the results highlighted recurrent themes amongst both patients and GDPs. These could be divided into the awareness of MRONJ, the burden of dental care, treatment planning strategies in this cohort of patients, and multidisciplinary co-ordinated care.

**Conclusion:**

Integration of dental services into oncology care remains an important aspect of BMA treatment for oncology patients. This article discusses relevant qualitative factors regarding the onco-dental interface and serves as a real-life template to optimise dental oncology care.

**Supplementary information:**

The online version contains supplementary material available at 10.1007/s00520-026-10951-0.

## Background

Bone-modifying agents (BMAs) are incorporated in routine cancer care to prevent skeletal-related events, treat hypercalcaemia, and reduce bone pain and cancer treatment-induced bone loss [1–3]. BMA treatment places patients at risk of developing medication-related osteonecrosis of the jaw (MRONJ). MRONJ is a rare oral complication occurring in 1–12% of patients with metastatic disease [4]. However, MRONJ is difficult to treat and causes significant morbidity to patients who may experience functional and psychological impacts on daily living [3, 5, 6]. The use of BMAs has risen significantly in recent years [6–8]. Denosumab prescription in the oncology setting has increased in the United Kingdom (UK) with an estimated 24.4% rise in National Health Service (NHS) expenditure between 2015/2016 and 2016/2017 [9]. The introduction of intravenous bisphosphonates in the treatment of early breast cancer also approximates a further 20,000 patients on bisphosphonates annually in the UK [10].

Current clinical guidelines recommend that patients achieve dental fitness, specifically the elimination or stabilisation of oral disease before commencement of MRONJ-implicated medications [5]. Accessing dental services to achieve these targets and maintaining this standard of oral care following BMA therapy remains challenging [7, 11]. Dental oncology pathways for high-risk oncology patients lack standardisation and effective incorporation into cancer care [12]. Patient self-reliance to engage with dental care remains the prominent pathway for patients to achieve dental fitness [13]. The prevention of MRONJ requires a multidisciplinary, collaborative approach by healthcare teams to ensure education of patients and promotion of high standards of oral hygiene and preventive dental measures [4–6, 14]. Preventative dental strategies are the most effective strategy to reduce the incidence of MRONJ and ensure optimal quality of survival for oncology patients [13, 15].

Awareness and provision of routine dental care for oncology patients is challenging for both patients and healthcare professionals [16]. Cohesion between medical and dental specialities can be lacking with resultant negative patient impacts [17–19]. A knowledge gap exists related to psychosocial impacts of BMA therapy, including MRONJ and its impact on quality of life [15]. It is important to understand the subjective dimensions of dental care in this oncology setting. Our study aims to investigate the attitudes of oncology patients about the importance of dental health during and after BMA therapy and to explore the attitudes of GDPs towards the provision of dental care for oncology patients undergoing BMA therapy.

## Methods

This was a qualitative study based on recorded semi-structured interviews, using a study-specific topic guide (appendices A and B), incorporating both focus groups and individual interviews. The recruitment included 10 oncology patients planned for BMA therapy, recruited from the oncology services in the Cork University Hospital, the South Infirmary Victoria University Hospital, and the Mercy University Hospital, alongside 20 GDPs who worked in both private practice and in the Cork University Dental School and Hospital (CUDSH).

The aims and methods of the study were explained to each participant both verbally and in written format. The voluntary nature of their participation was emphasised, and written consent was obtained. Patient participation (*n* = 10) included one focus group (*n* = 6). Individual telephone interviews (*n* = 4) were also conducted to facilitate individual patient requests. GDP participation included three focus groups (*n* = 6), (*n* = 6), and (*n* = 8). Each focus group was mixed with both age and gender alongside oncology diagnosis or dental special interest. Focus groups were conducted in a non-clinical setting in Cork University Dental School by a trained facilitator (first author). A relationship was established prior to the commencement of the qualitative study, and there were no drop-outs from the study. Semi-structured interview questions are attached with topic guides (see appendices A and B) for patient and GDP cohorts. Focus groups and telephone interviews ranged from 10 to 60 minutes in timeframe. There were no repeat interviews performed. The focus groups and telephone individual interviews were digitally recorded, transcribed (*GoTranscript*) and anonymised. The transcripts were analysed on a line-by-line basis, and thematic analysis was conducted using the six-phased recursive method. The data was analysed, and a coding system was devised to link passages of descriptive themes and opinions within the text. One researcher (first author) coded the interviews which were analysed to develop encompassing descriptive narratives, defining themes, and report generation. The authors appreciate the process of reflexivity throughout the conduction of this research project. The reflexivity process prompted the authors to critically reflect on their subjective role and how their perspective shaped the analysis. These biases are accepted given the researcher’s inherent involvement with the process, data collection, and analysis of data.

**Fig. 1:**
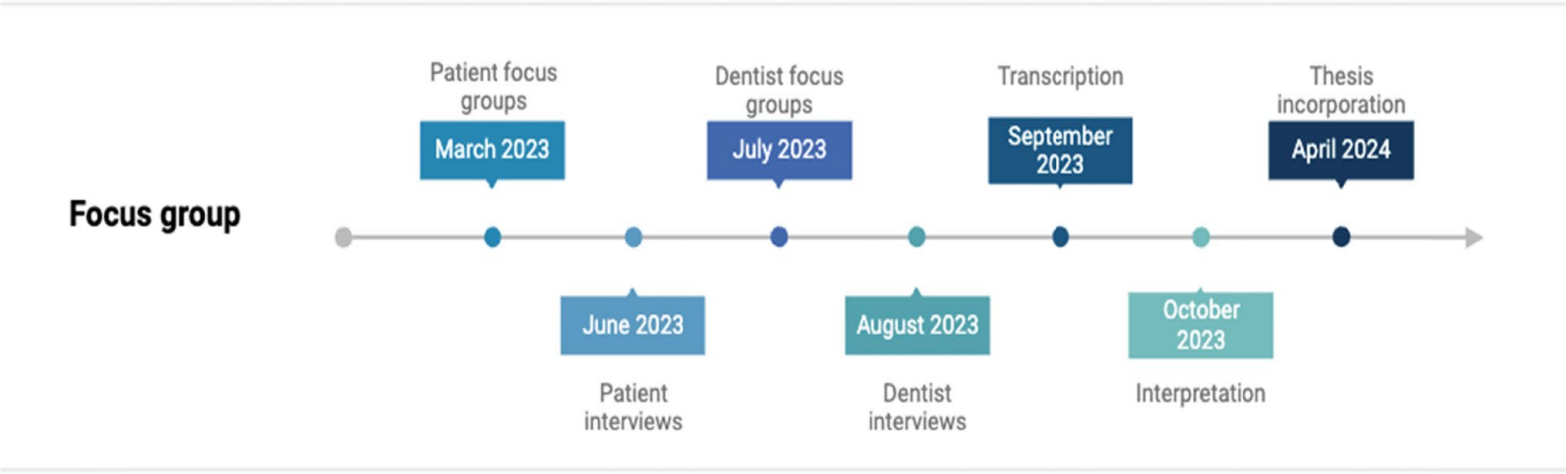
The timeframe of the study

The ethical approval application was granted by the Clinical Research Ethics Committee (CREC), University College Cork (UCC) under the title “Oral Health Status and Dental Treatment Needs of Oncology Patients Receiving Bone-Modifying Agents”. The stakeholders included UCC and Health Service Executive (HSE). The study was conducted in accordance with the requirements of the WMA Declaration of Helsinki (2008). The study timeline is demonstrated in Fig. 1.

### Patient inclusion criteria

Ten oncology patients participated in one focus group (*n* = 6). Telephone interviews were conducted with four patients due to patient preference and privacy (Table 1). Their BMA therapy was prescribed for oncology-related indications only, including bone metastases, bone pain, hypercalcaemia, and prevention of bone metastases in breast cancer patients. Oncology patients with early or metastatic disease were included. Their BMA regime was prescribed at oncology dose regimes, including intravenous administration of bisphosphonates or subcutaneous receptor activator of nuclear kappa beta ligand (RANK-L) inhibitors. Exclusion criteria included patients who had previous exposure to a BMA for either non-oncological or oncological purposes. Patients were also excluded from the study who did not wish to participate or who wanted to proceed with treatment from their own general dental practitioner.

#### GDP inclusion criteria

The GDP cohort included 20 staff members of the CUDSH who were also actively practising dentistry in general dental practice. Three focus groups took place (*n* = 6, *n* = 6, and *n* = 8) in the CUDSH where dentists expressed their opinions and experiences of treating patients who received BMA therapy in general dental practice (Table 2).

## Results

Tables 1 and 2 summarise the patient and GDP cohorts in this study.

**Table 1:** The oncology patient cohort in both the focus group and telephone interviews

Form of interview	Patient number (*P*)	Age	Sex	Oncology diagnosis (*stage)	Oncology treatment	BMA therapy
Focus group (FG)	P1	49	Male	Prostate cancer (4)	Abiraterone, androgen deprivation therapy	Zoledronic acid
	P2	61	Female	Breast cancer (2)	Surgery and chemotherapy	Zoledronic acid
	P3	61	Female	Breast cancer (2)	Surgery	Denosumab
	P4	65	Male	Prostate cancer (4)	Abiraterone, androgen deprivation therapy	Zoledronic acid
	P5	69	Male	Prostate cancer (4)	Abiraterone, androgen deprivation therapy	Zoledronic acid
	P6	67	Female	Breast cancer (4)	Surgery, hormone replacement therapy, radiation therapy	Zoledronic acid
Telephone interview (TI)	P7	62	Female	Breast cancer (2)	Surgery, hormone replacement therapy	Zoledronic acid
	P8	50	Male	Metastatic sarcoma (4)	Chemotherapy	Zoledronic acid
	P9	53	Female	Breast cancer (2)	Surgery, chemotherapy, radiation therapy	Zoledronic acid
	P10	61	Female	Breast cancer (4)	Surgery, chemotherapy	Zoledronic acid

**Table 2:** The general dental practitioner cohort in the three focus groups

Form of interview	Dentist number	Age	Sex	Year since qualification	Dental speciality/special interest
Focus group					
Focus group 1	D1	43	Female	19	General
	D2	59	Male	37	General
	D3	36	Female	13	General/prosthodontics
	D4	60	Male	37	General
	D5	53	Male	29	General
	D6	28	Female	5	General
Focus group 2	D7	40	Male	17	General/endodontics
	D8	54	Female	31	General
	D9	38	Female	5	General
	D10	60	Male	38	General
	D11	37	Female	13	General/prosthodontics
	D12	54	Female	31	General
Focus group 3	D13	55	Female	33	General/prosthodontics
	D14	31	Female	5	General/oral surgery
	D15	44	Female	21	General/endodontics
	D16	59	Female	35	General
	D17	44	Female	21	General
	D18	63	Female	40	General
	D19	43	Male	11	General
	D20	30	Female	6	General

Table 1 highlights patients who are planned for BMA therapy (*N* = 10), which included patients with metastatic prostate cancer (*N* = 3), early-stage breast cancer (*N* = 4), late-stage breast cancer (*N* = 2), and metastatic sarcoma (*N* = 1).

Table 2 highlights the GDP participants in this study (*N* = 20) who were working both in the CUDSH and in private practice. The scope of practice included general dental practice (*N* = 14), oral surgery (*N* = 1), endodontics (*N* = 2), and prosthodontics (*N* = 3).

Analysis of data highlighted four key themes in relation to oncology patients and GDP experiences with BMA therapy in the oncology setting:

- MRONJ awareness
- Dental neglect
- Treatment planning-related decisions
- Onco-dental service integration

### Theme 1: MRONJ awareness

There was clear evidence that MRONJ was not an informed component of the oncology patient’s journey. Input from patient transcriptions highlights the lack of education and knowledge of MRONJ in this cohort prior to BMA therapy. Participants expressed the poor integration of dental advice prior to and following BMA therapy, which added to the burden of care. Self-reliance and consequential treatment anxiety were experienced by patients (see Appendix A). Dentists reiterated the lack of professional awareness and knowledge of MRONJ, particularly when confronted with suspected MRONJ cases. The importance of MRONJ education was highlighted as an important aspect of care, and the appreciation of education was acknowledged by patients. Dentists also expressed concerns about treating patients who had historic BMA exposure or transitioning from oral to intravenous bisphosphonates (see Appendix A). Concerns such as a rising prevalence of MRONJ and incidence of BMA therapy prescription worried dentists.

### Theme 2: dental neglect

The theme of poor dental adherence was also evident as patients explained about their previous dental motivation and lack of dental homes. Dental disease and the chronic effects of poor dental care dictated their motivation for dental care and engagement following BMA administrations.

**Fig. 2:**
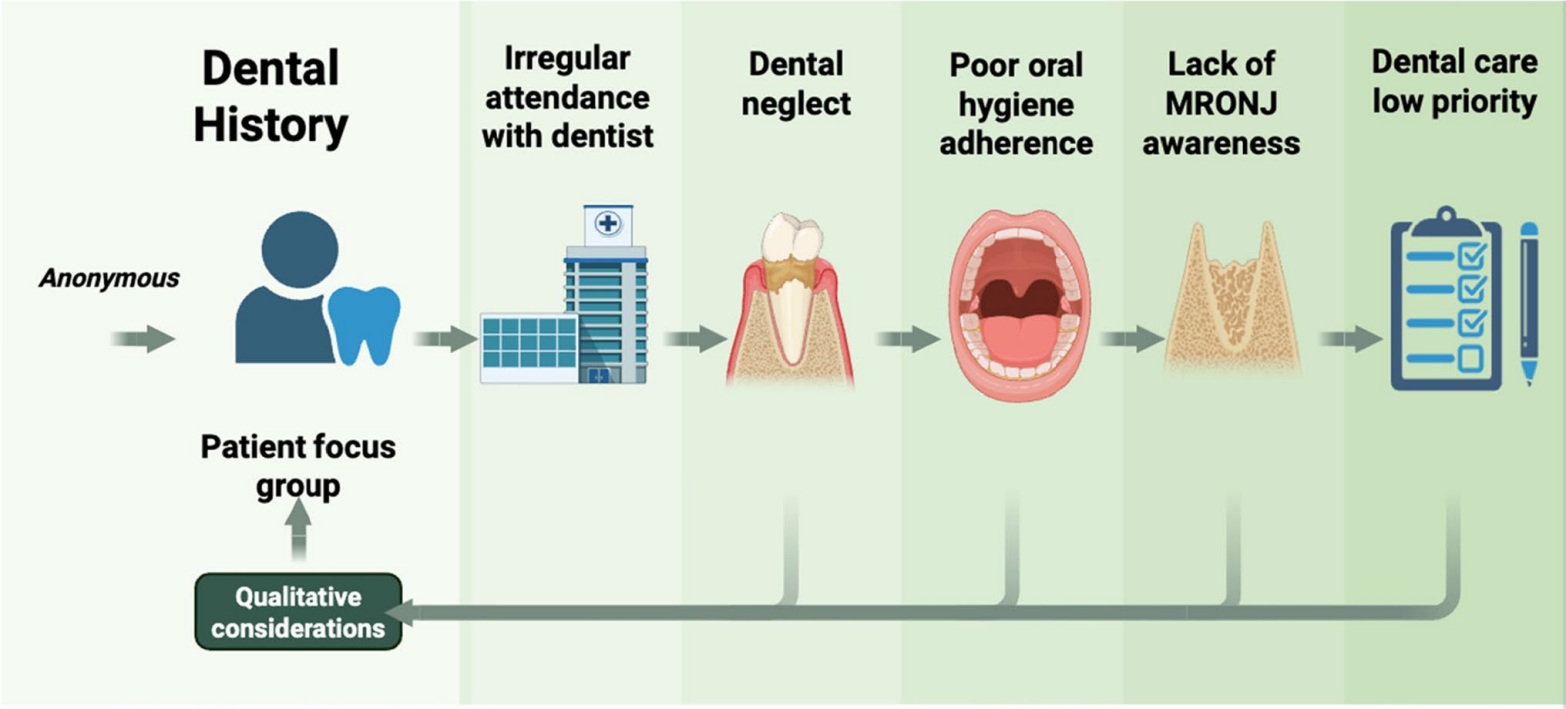
Some key topics of interest from a patient’s dental history

Other important aspects of dental neglect included poor attendance histories, dental anxiety, poor oral hygiene regimes, and the insignificance of dental care in patients’ lives (see Appendix A). Attendance at a dentist was largely regarded on an emergency basis, with negative previous dental experience dictating their future engagement with a dentist (Fig. 2).

### Theme 3: treatment planning-related decisions

The challenges of dental fitness and the parameters that define dental fitness remain vague and are often dictated by individual and variable opinions. Treatment planning, tooth prognosis, and co-ordination of dental care present challenges for the dental profession. Often, dental disease such as periodontitis is a long-standing, chronic, inflammatory condition that cannot be resolved prior to dental fitness (see Appendix A).

**Fig. 3:**
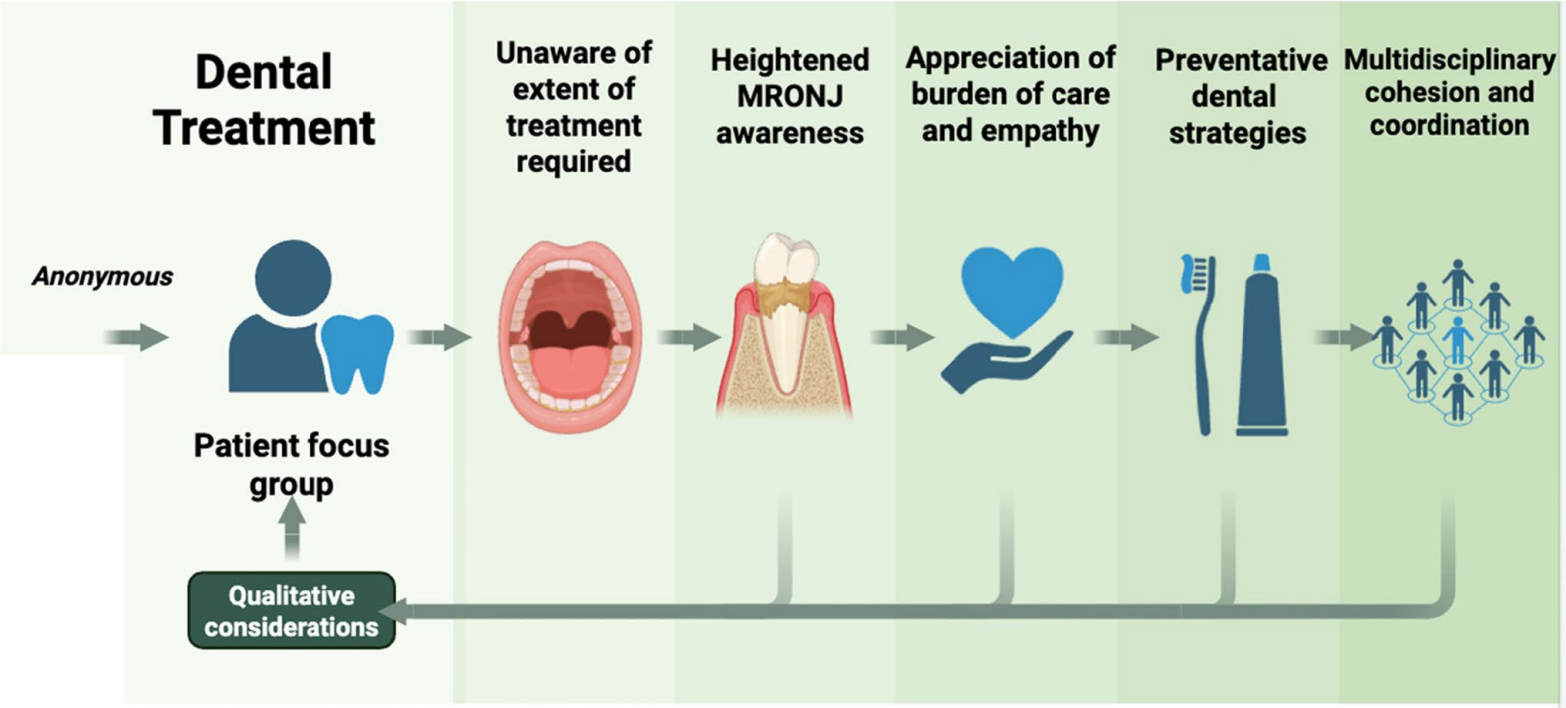
Some patient-dental treatment-related challenges

Dentists also expressed concerns about the transient nature of dental fitness and that currently there is a deficiency of guidance to help regulate dental fitness. The nature of teeth with a questionable prognosis or chronic disease such as periodontal disease challenges dental treatment plans (see Appendix A). Financial restraints, including limitations of medical card treatment capacity or the feasibility to remain under the additional long-term care of a hygienist, can pose financial challenges to patients and limit treatment plans. The burden of care for oncology patients was also mentioned as an additional stressful factor along their journey. Importance factors such as empathy, quality of life considerations for patients amidst their dental treatment, and understanding the psychological impact of sometimes invasive dental treatment within a limited timeline influenced the patient’s dental experience (Fig. 3).

### Theme 4: onco-dental service integration

**Fig. 4:**
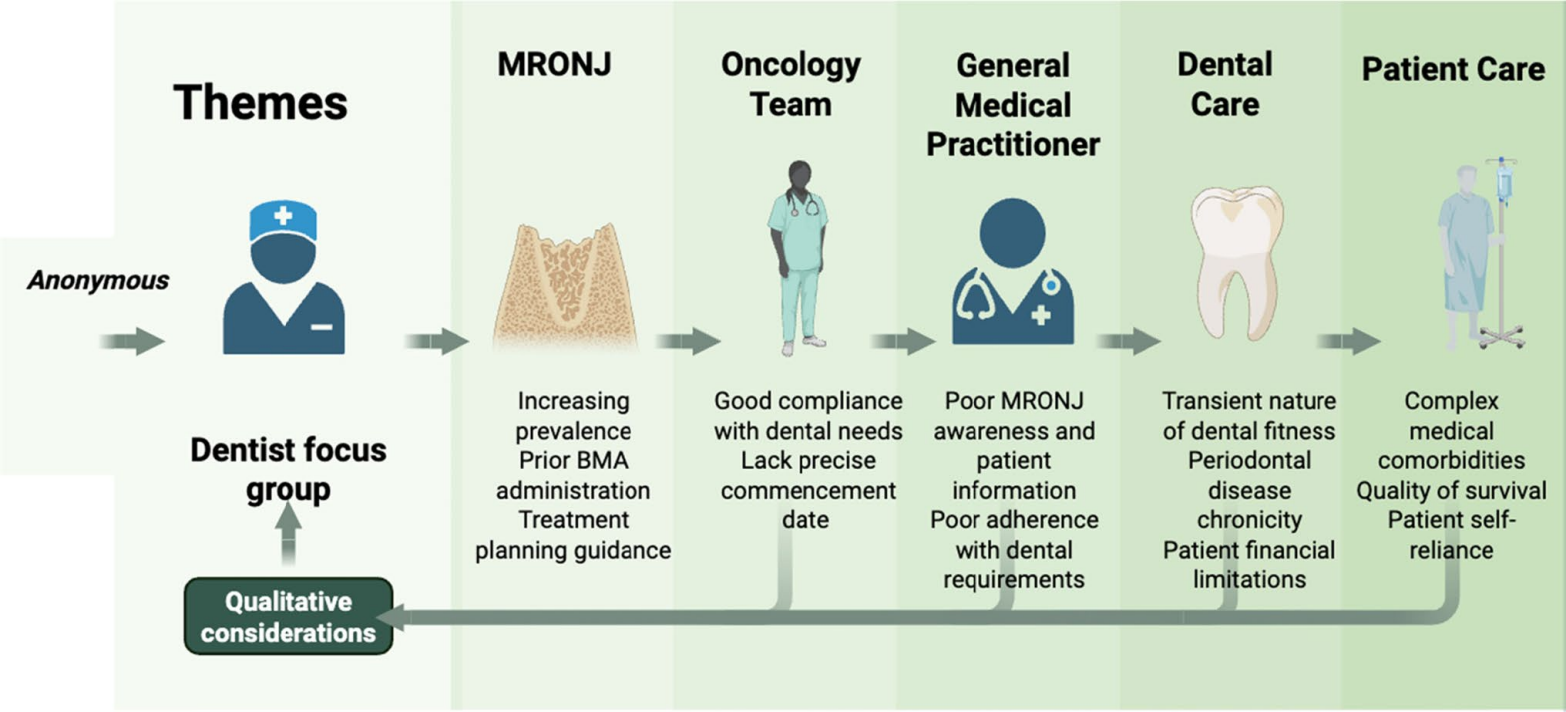
Important themes which emerged from the dentist focus groups, in particular the challenges that present to dental practice with patients on BMA therapy

The burden of dental treatment amidst oncology care is demanding, and patients remarked on the impact of this additional facet of care. The importance of multidisciplinary-driven care was considered important by patients. Cohesion of care amongst professionals remains an important aspect of oncology care for patients (see Appendix A). Our analyses revealed recurrent themes discussed by patients regarding dental treatment. Patients expressed concerns about their lack of awareness of MRONJ, and confidence in a collaborative oncological approach to dental care was reassuring. Cohesion of local onco-dental services and ease of communication with oncology nurses and coordinators remained important for patients. The burden of dental treatment, including the intense period to achieve dental fitness, the scale of dental treatment required at times, the psychological burden of dental care and the change in oral functional capacity as a result of extractions, remains worrisome for patients (Fig. 4). Information such as printed leaflets and written information was valuable, and dental staff empathy and understanding remained a recurrent positive theme during the dental treatment process.

## Discussion

Our study investigated the attitudes for both oncology patients prior to BMAs and GDPs treating this cohort of patients. We uncovered some interesting themes such as the burden of care for patients to access dental services, the lack of awareness of MRONJ, gaps that co-exist between dental and oncology services alongside treatment planning challenges, and lack of guidance-based information for GDPs. Thematic analysis assisted the development of these themes, which identified deficiencies within the current structures of dental care for these patients [11]. Adequate incorporation of dental oncology services for patients prior to BMA treatment remains sparse and unstructured [20]. Albeit MRONJ remains an uncommon clinical consequence of BMA treatment, associated morbidity and quality of life limitations are significant [15]. A retrospective review of 15,357 oncology patients who received BMAs revealed 1706 patients (11.1%) underwent a dental assessment prior to BMA treatment [21]. This research has documented the increased uptake of dental involvement prior to BMA treatment from 4.4% in 2007 to 16.7% in 2019; 12.5% of this cohort subsequently developed MRONJ [21]. A multidisciplinary approach to combat and prevent MRONJ remains the most viable option for BMA therapy patients due to the effectiveness of education, preventative dental regimes, and pre-therapeutic dental treatment in the reduction of MRONJ [4, 13]. The effects of cancer-induced therapies remain prominent, including the risk of MRONJ [12, 22, 23]. Qualitative studies have highlighted recurrent themes for management of patients with MRONJ or at risk of developing MRONJ [17]. Quality of life factors, interprofessional management, and the wider context of oral and healthcare provision are recurrent topics for patients at risk of MONRJ [17]. This study is the first study using qualitative methods to report both patient and dental professional attitudes to bone-modifying agents in the oncology setting.

Patient reflections included both positive and negative themes surrounding the care of premedication dental care in the oncology setting. This study highlights the importance for both patient and clinician of the value of cohesion within dental and oncology services, evidence-based holistic care, and access to oncology and dental team members. BMAs remain prominent in anti-cancer therapies; however, quality of life studies and advancements in qualitative aspects of BMA therapy are lacking [24]. Patient-related factors which posed the greatest challenge to dental care included symptomatic-driven care, no consistent dental home, dental anxiety, dental neglect, and poor oral hygiene habits, which all negatively impacted active engagement with a dentist at the time of BMA treatment.

Awareness of the risk of MRONJ following BMA therapy in the wider healthcare community is heterogenous [16, 25–27]. Over-reliance on patient self-reliance and the lack of standardised pathways for dental provision within oncology frameworks increase the burden of care and potential complications of anti-cancer therapy [13, 28]. Unification of healthcare professional utilities such as information leaflets and optimisation of knowledge sharing such as designated dental oncology referral forms and dental oncology discharge forms (appendices C and D) promote effective multidisciplinary engagement and optimise preventative-driven dental strategies for these patients [4, 13, 29, 30]. Although our research did not include the attitudes of pharmacists in relation to BMA therapy, the literature has reported the additional benefits of the pharmacist as a relevant source of medication information, who is another aspect of holistic care within dental oncology strategies [17, 31].

Emergence of qualitative themes amongst dental professionals included treatment planning challenges, dental fitness parameters, management of the emergency patient following BMA treatment, and the fluctuant nature of chronic periodontal disease. The cohort of dentists in this study (*n*= 20) noted the significance of psychological and functional burdens to patients during their dental treatment. Communication and interdisciplinary relations were also highlighted as a valuable component of the onco-dental interface. A study by Raj et al. (2016) analysed the communication among general medical practitioners, dentists, and pharmacists regarding MRONJ, showing that important communication targets could vary depending on each country’s medical system [32]. The use of focus groups and telephoneinterviews optimises the development of qualitative themes and highlights areas of concern amongst patients and dentists. The questionnaires used in this study were developed from previously validated questionnaires from the literature which consider the importance of reliability and validity of the material used throughout this research [17, 25, 33–37].

Mechanisms of reducing both perceived and actual professional isolation and improving collaborative care and mechanisms of communication between professions should also be reviewed. The house of care model has been used previously in other spheres outside of dentistry, which provides a framework for patient-centred coordinated care which relies on four key components: (1) engaged and informed individuals, (2) professionals committed to partnerships, (3) organisational and supporting processes, and (4) system wide approaches to commissioning [17, 38]. Cross-pollination and application of valuable tools such as care models may help guide dental provision for this group of patients [38]. The integration of oral healthcare into the wider healthcare system following this model could potentially address the issues identified in our research, optimise prevention of MRONJ, and address other areas in which oral health impacts the overall health and well-being of patients [39].

Important themes emerged from the dentist focus groups, in particular the challenges of treatment planning and treating patients on BMA therapy. Discrepancies remain in evidence-based guidance protocols, and national funding for oral health is fluctuant [40]. Streamlining patients at risk of MRONJ remains inconsistent and unstructured for oncology services, particularly as preventative strategies bear little significance in overall oncology regimes. The National Cancer Care Programme has endorsed a baseline assessment form for baseline patient parameters, including oral health. The vast majority of MRONJ containing guidelines and documents originate from the dental community and often lack unity with oncology strategies. The first clinical guidance for MRONJ was published in 2005 by the American Academy of Oral Medicine to promote preventative and management strategies for MRONJ [41]. Since then, organisations such as the Scottish Dental Clinical Effectiveness Programme (SDCEP) [42], Cochrane Oral Health [43], MASCC/ISOO/ASCO Clinical Practical Guidance [6], American Association of Oral and Maxillofacial Surgeons (AAOMS) [5], Italian Societies of Maxillofacial Surgery and Oral Pathology and Medicine/Italian Allied Committee on ONJ (SICMF/SIPMO) [44, 45], Royal College of Physicians London [46], and Workshop of European Task Force on MRONJ [47] have published documentation in the literature to guide the management of MRONJ. The collective themes from the study summarise an inherent lack of cohesion and high-quality evidence-based practice. Unfortunately, there remains a lack of unity surrounding the treatment and management of patients at risk of MRONJ, which is translatable for patients and clinicians through the emergence of themes 1–4. Such examples of these discrepancies include use of antibiotics and management strategies of MRONJ; MASCC/ISOO/ASCO reserve surgical interventions for refractory MORN compared to the SICMF/SIPMO and Workshop of European Task Force on MRONJ advocate early surgical intervention for all stages of MRONJ and optimal primary mucosal closure [12, 45, 47]. The Cochrane report does not provide any definitive surgical guidance for the management of MRONJ [43]. Other important aspects of discussion include treatment planning for patients on BMA therapy, the role of antibiotics therapy in the management of MRONJ, the impact of smoking and systemic disease, and MRONJ medical management strategies, which require further high-quality research to unify guidance documents [5, 8, 47]. Guidance documents have reached a consensus about the importance of preventative dental strategies in patients at risk of MRONJ, the importance of 3–6 monthly dental visits, and the early detection of MRONJ [5, 12, 43].

There were some limitations in this study which only captured a small proportion of patients and GDPs at a single site in the Republic of Ireland, which creates challenges when applying the results to the wider population. The study was held during the COVID-19 pandemic which may have influenced dental engagement prior to recruitment. This may influence the perception of dental care, particularly in the patient cohort regarding frequency of attendance with a dentist and their overall dental treatment needs. The dental hospital was co-located with one of the sources of referrals which was noted as a unique situation nationally compared to community and larger oncology referrals centres.

## Conclusion

This is the first study to examine both patient and dentist attitudes to oncological BMA therapy in the dental setting. Focus groups and telephone interviews allowed us to identify notable gaps in awareness, education, and motivation to optimise anti-cancer therapy for patients and dentists. This qualitative data demonstrates the importance of prophylactic dental integration instead of reflexive intervention in the dental management of oncology patients on BMA therapy.

## Supplementary information

Below is the link to the electronic supplementary material.

ESM 1 (DOCX 16.9 KB)

ESM 2 (DOCX 338 KB)

## Data Availability

No datasets were generated or analysed during the current study.
