# Supplementary material for: A qualitative analysis of dental challenges for oncology patients receiving bone-modifying agents
Source: Support Care Cancer. 2026 Jul 24;34(8):800. doi: 10.1007/s00520-026-10951-0 (PMC13400693; doi:10.1007/s00520-026-10951-0)
Supplement: Supplementary file 1 — (DOCX 16.9 KB) [file 520_2026_10951_MOESM1_ESM.docx]

Appendix A – Patient and clinician experiences relating to themes 1-4.

Theme 1: MRONJ awareness

*“No, I wasn’t aware until I went to you – and I wasn’t even sure why I was being sent to you; I hadn’t really looked it up. But then you explained the link with the Zometa and the dental issues”, (P4, FG).*

*“They usually have… they come to you for… they don’t know why they’re turning up. Doctors need a letter, like, and then why. But the risk of osteonecrosis is played down”, (D14, FG3).*

Theme 2: dental neglect

*“And taking all my teeth out, but you really opened my eyes, you really did – cleaning them and all that kind of stuff. I wash my teeth two or three times a day anyway, but thinking… I thought my teeth were grand, they were fairly bad, and I was a kind of nervous”, (P6, FG).*

*“That’s all I thought. I never knew that there was dental care involved. And you know yourself; I got an awful shock when you told me I’d have to have three teeth removed. And you’re only entitled to one visit – so, three on the day; I just couldn’t. It’s a lot to have to deal with”, (P8, TI).*

*“I couldn’t tell you that now, being honest, I never was keen to go to the dentist. I had no real interest in looking after my teeth, which I should have, really. But I never did, being honest”, (P5, FG).*

Theme 3: treatment planning-related decisions

*“I think the problems start when you get a letter saying can you give us a cert or whatever, to say that they’re dentally fit – but it’s very hard… I always found that very difficult to predict. So, I don’t know if there were guidelines to guide treatment, but the guidelines aren’t there, you don’t want to be too aggressive either. It's a balance”, (D13, FG3).*

*“Definitely that’s lacking (guidance). And then you’re often caught in this awkward position then when they come in, they’re in agony with a tooth, they’ve been taking this drug for however long”, (D16, FG3).*

*“I think, the perio ones are the problem ones. If they’ve got reasonable perio, and say a tooth blows up, you can root canal it. And I’ll say, look, we’ll have to root canal that now, we can’t be taking that out. But if they’ve got the grade one and grade two mobile, and if they’ve got a denture in there that’s dependent on it or something, should it go, or we’re going to have to make another denture for it – that’s where it gets complicated really, I think, because you are making a major change in the mouth there”, (D10, FG2).*

*“It does put you under a lot of pressure to do it, and to decide what’s best to do. In my opinion is, if it’s any way borderline, in my opinion to remove the tooth”, (D2, FG1).*

*“I see it increasingly in practice, all the time. There’s obviously the oral ones, we see loads of and the IV version of it, obviously the ones that are most at risk. But we do see it, and more and more, for sure. You see more patients coming in after starting, and that haven’t had assessments. A lot of that though is the oral ones, not the IV ones. But often it has happened, where people come in after they’ve started”, (D4, FG1).*

*“I would say that when it comes to oncology side of things, it’s normally that they were in trouble, because generally there’s such a rush for the patients to get on the treatment, there’s so many other things happening, that the dentist will fall to the bottom of the list”, (P2, FG).*

Theme 4: onco-dental service integration

*“Like, they’re going through an awful traumatic time; a lot of them have cancer treatments – and then to have to tell them that they’re going to have all of these teeth removed, it’s a double-whammy for them. But some communication with the consultant is nice to have, and I suppose when we’re writing these letters we’ll say, at this moment in time, which is all you can do”, (D17, FG3).*

*“It wasn’t really – I went through it no problem. I was delighted with the service. The nurse, put me onto you up here and got them all done – that was it, I was delighted”, (P10, TI).*
