# Supplementary material for: A qualitative analysis of dental challenges for oncology patients receiving bone-modifying agents
Source: Support Care Cancer. 2026 Jul 24;34(8):800. doi: 10.1007/s00520-026-10951-0 (PMC13400693; doi:10.1007/s00520-026-10951-0)
Supplement: Supplementary file 2 — (DOCX 338 KB) [file 520_2026_10951_MOESM2_ESM.docx]

**Appendices:**

**Appendix A**

**Patient topic guide**

# **Moderator Introduction and Purpose of the Interview (2 minutes)**

Hello. My name is Harriet. I’d like to start off by thanking you for taking time to participate today. We’ll be here for about forty-five minutes.

The reason we’re here today is to gather your opinions about your knowledge of MRONJ and experience of dental care before receiving your intravenous cancer therapy.

I’m going to lead our discussion today. I will be asking you questions and then encouraging and moderating our discussion.

I would like you to know that this interview will be voice recorded. The identities of participants will remain confidential. The recording allows us to revisit our discussion to ensure we have interpreted your comments correctly and the results can then be used for developing research papers and presentations.

# **Ground rules (2 minutes)**

To allow our conversation to flow more freely, I’d like to go over some ground rules.

1. Everyone doesn’t have to answer every single question
2. This is a confidential discussion in that I will not report your names or who said what to anyone. Names of participants will not even be included in the final report about this meeting. It also means, except for the report that will be written, what is said in this room stays in this room.
3. We stress confidentiality because we want an open discussion.
4. There are no “wrong answers,” just different opinions. Say what is true for you, even if you’re the only one who feels that way.
5. Let me know if you need a break.
6. Are there any questions?

# **Interview Questions (35-45 minutes)**

- 1. Personal history and knowledge about dental care
     1. How often do you attend your dentist?
     2. Is dental care an important to you?
     3. Did you know about the mouth complications of your IV cancer treatment?

1. Dental care experience prior to intravenous (IV) cancer therapy
   - 1. Are you aware you require a pre-therapy dental examination and necessary treatment before your IV cancer therapy?
        1. Who informed you about your basic dental assessment?
        2. How did you access a dental service?
        3. What difficulties did you experience in the process of accessing a dental service? Availability restrictions? Financial restrictions? Travel? Aftercare support?
        4. Did you attend the dental assessment and if not, why so? Travel? Anxiety? Did not feel it was necessary?
2. Basic treatment requirements
   - 1. What treatments did you receive?
     2. Satisfaction with treatment
        1. Was it successful?
        2. What do you understand about success of the dental treatment?
        3. Are you aware of classic signs and symptoms of MRONJ?
3. Treatment outcomes
   - 1. Do you have a long-term review regime in place?
        1. Do you consider regular dental reviews necessary while taking you IV cancer therapy?
     2. Has the process of attending a dental review prior to IV cancer therapy influence your oral health?
4. Has the process of a dental assessment prior to IV cancer therapy increased your awareness of MRONJ?

# **Closing**

Thanks for coming today and talking about your knowledge of MRONJ and experience of pre-therapy dental assessments. Your comments have given us lots of valuable information. I thank you for your time.

**Appendix B**

**Dentist topic guide**

# **Moderator Introduction and Purpose Interview (2 minutes)**

Hello. My name is Harriet. I’d like to start off by thanking you for taking time to participate today. We’ll be here for about 45 minutes.

The reason we’re here today is to gather your opinions about your knowledge of MRONJ and experience of treating this cohort prior to bone modifying agents (BMAs).

I’m going to lead our discussion today. I will be asking you questions and then encouraging and moderating our discussion.

I would like you to know that this interview will be voice recorded. The identities of participants will remain confidential. The recording allows us to revisit our discussion to ensure we have interpreted comment correctly and the results can then be used for developing research papers and presentations.

# **Ground rules (2 minutes)**

To allow our conversation to flow more freely, I’d like to go over some ground rules.

1. This is a confidential discussion in that I will not report your names or who said what to anyone. Names of participants will not even be included in the final report about this meeting. It also means, except for the report that will be written, what is said in this room stays in this room.
2. There are no “wrong answers,” just different opinions. Say what is true for you, even if you’re the only one who feels that way.
3. Let me know if you need a break.
4. Are there any questions?

# **Interview Guide (35-45 minutes)**

- 1. Personal history and knowledge of patients prior to receiving a BMA?
     1. Are you aware of bone modifying agents?
     2. Have you treated a patient prior to their administration?
     3. Did you know about the oral complications of BMAs?

1. Dental care experience prior to intravenous (IV) cancer therapy
   - 1. Are you aware this cohort require a pre-therapy dental examination and basic dental treatment before their IV cancer therapy?
        1. Who informed you about their basic dental requirements?
        2. How did they access your dental service?
        3. What difficulties did you experience while treatment planning and treating this cohort?
        4. Did you consider this treatment planning process beyond you scope of capabilities?
        5. How do you educate you patient on the oral complications of BMAs?
2. Basic treatment requirements
   - 1. What treatments did they receive?
     2. Satisfaction with treatment
        1. Was it successful?
        2. What do you understand about success of the standard dental treatment in this cohort?
        3. Are you aware of the presentation of MRONJ?
        4. What protocol do you adhere to with regard to the management of MRONJ?
3. Treatment outcomes
   - 1. Do you have a long-term review regime in place for these patients?
     2. What resources do you recommend to you patients for further education regarding BMAs and oral complications?
4. Has the dental profession provided additional CPD courses or resources to manage this cohort of patients?

# **Closing**

Thanks for coming today and talking about your knowledge of MRONJ and experience treating this cohort prior to BMA therapy. Your comments have given us lots of valuable information. I thank you for your time.

**Appendix C - Dental oncology referral form:**

**
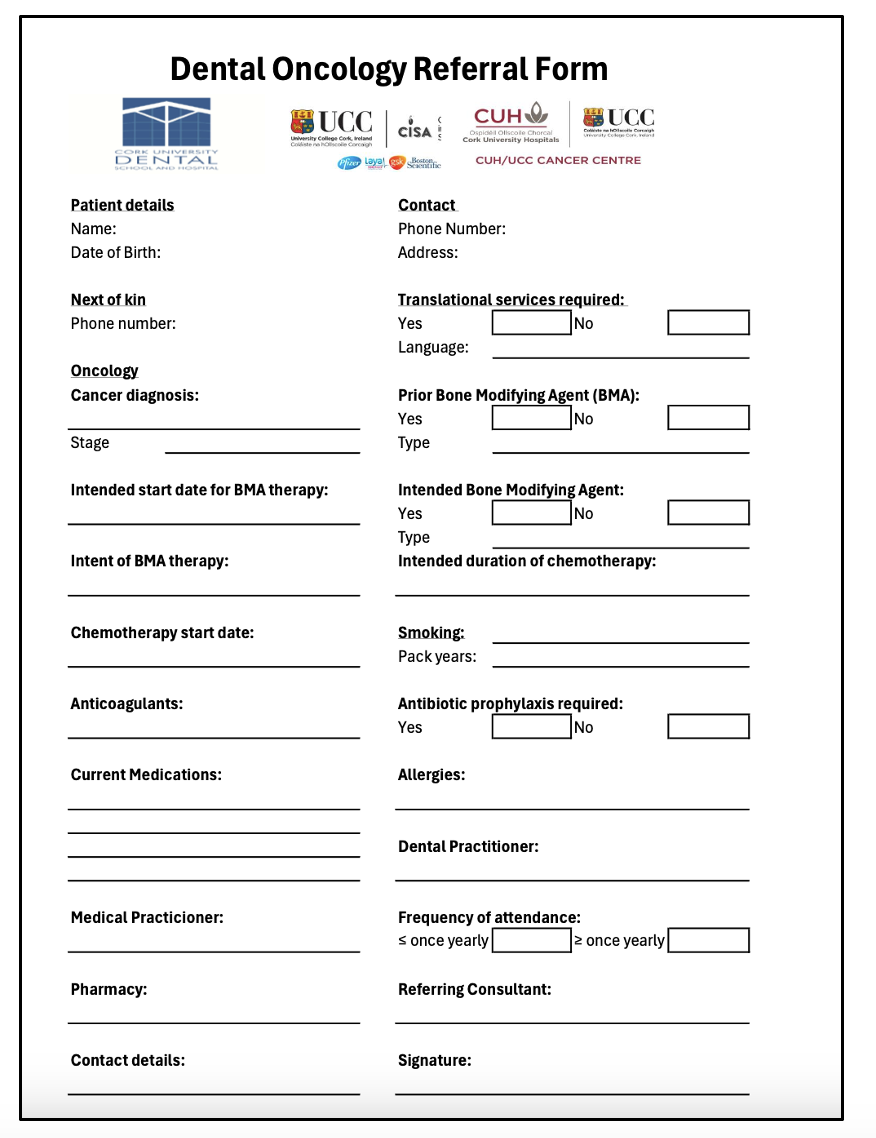
**

**Appendix D - Dental oncology discharge form:**

**
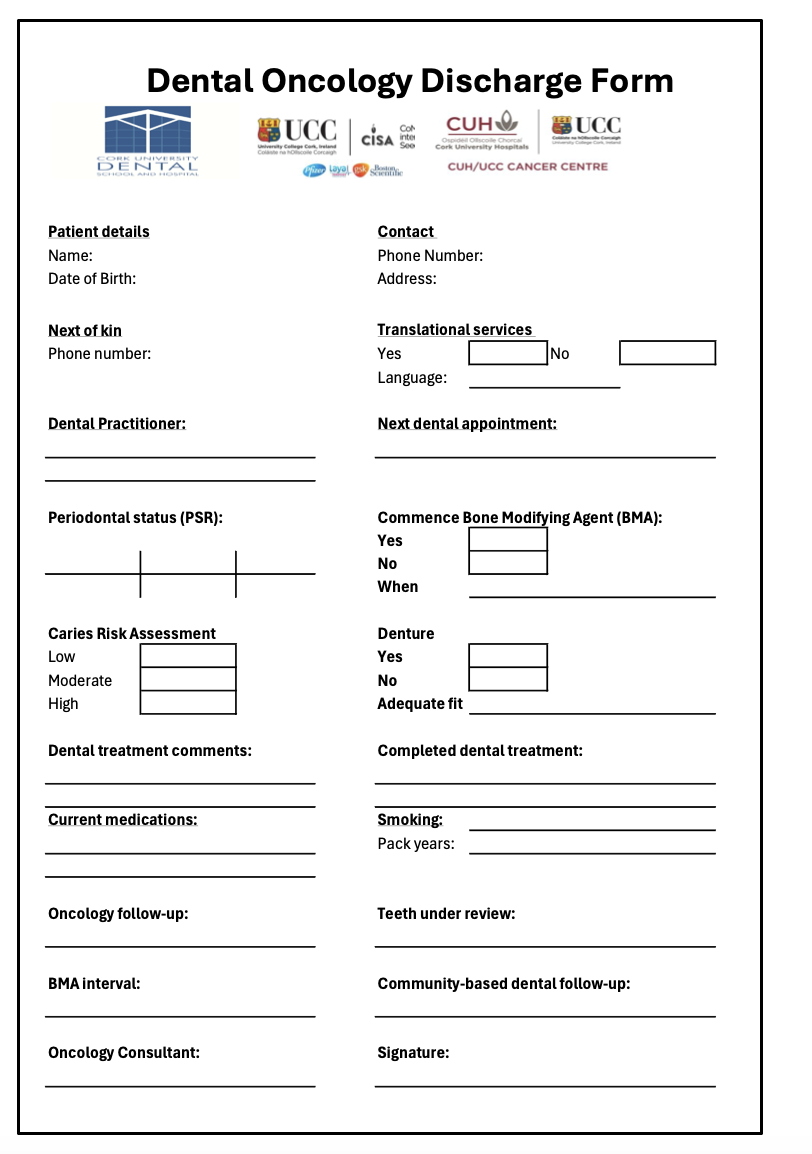
**
